# Identification of noncoding RNA expression profiles and regulatory interaction networks following traumatic spinal cord injury by sequence analysis

**DOI:** 10.18632/aging.101919

**Published:** 2019-04-18

**Authors:** Wenzhao Wang, Yanlin Su, Shi Tang, Hongfei Li, Wei Xie, Jianan Chen, Lin Shen, Xinda Pan, Bin Ning

**Affiliations:** 1 Jinan Central Hospital Affiliated to Shandong University, Jinan, China; 2 Department of Orthopaedics, West China Hospital, Sichuan University, Chengdu, China; 3 Department of Radiology, West China Hospital, Sichuan University, Chengdu, Sichuan, China; 4 Department Emergency Medicine, Affiliated Hospital of Taishan Medical University, Taian, China

**Keywords:** traumatic spinal cord injury, noncoding RNA, competing endogenous RNA, axonal regeneration

## Abstract

Aim: To systematically profile and characterize the noncoding RNA (ncRNA) expression pattern in the lesion epicenter of spinal tissues after traumatic spinal cord injury (TSCI) and predicted the structure and potential functions of the regulatory networks associated with these differentially expressed ncRNAs and mRNAs.

Results: A total of 498 circRNAs, 458 lncRNAs, 155 miRNAs and 1203 mRNAs were identified in TSCI mice models to be differentially expressed. The regulatory networks associated with these differentially expressed ncRNAs and mRNAs were constructed.

Materials and methods: We used RNA-Seq, Gene ontology (GO), KEGG pathway analysis and co-expression network analyses to profle the expression and regulation patterns of noncoding RNAs and mRNAs of mice models after TSCI. The findings were validated by quantitative real-time PCR (qRT-PCR) and Luciferase assay.

Conclusion: noncoding RNAs might play important roles via the competing endogenous RNA regulation pattern after TSCI, further findings arising from this study will not only expand the understanding of potential ncRNA biomarkers but also help guide therapeutic strategies for TSCI.

## INTRODUCTION

Spinal cord injury (SCI) is a disabling neurological condition with high economic and social costs; SCI is characterized by the loss of neural tissue and consequent deficits in sensory and motor functions [1]. Each year, half a million people damage their spinal cord, and the injury is almost always life-changing [2]. SCI increases the risk of involuntary movements, bladder and gastrointestinal disorders, and depression [1]. SCI can also be caused by iatrogenic procedures, infection, vascular lesions or tumors, but the most common cause is trauma [3]. Traumatic SCI (TSCI) causes cell necrosis, the disconnection of surviving neurons, and the irreversible interruption of ascending and descending neurotransmission [4]. Unfortunately, recent studies have demonstrated that no effective treatments exist for achieving complete neurological or functional recovery after TSCI. Moreover, the key mechanisms governing the cellular response to injury are largely unknown [5]. A better understanding of the cellular and molecular mechanisms following TSCI is necessary to develop new strategies to promote axonal regeneration and functional recovery.

Noncoding ribose nucleic acids (ncRNAs), which are a class of genetic, epigenetic and translational regulators, have been found to play key roles in various physiological and pathological processes [6]. No less than 70% of the human genome is transcribed, but protein-coding transcripts account for no more than 2%, and extensive transcripts derived from most of the genome generate a large proportion of ncRNAs [7]. Theoretically, ncRNAs do not encode proteins but instead functionally regulate the translation of proteins and can be classified into two types: housekeeping ncRNAs, which consist of small nucleolar RNAs (snoRNAs), small nuclear RNAs (snRNAs), rRNAs, and tRNAs; and regulatory ncRNAs, which consist of microRNAs (miRNAs), long ncRNAs (lncRNAs) with a relatively flexible length of >200 nucleotides, and circular RNAs (circRNAs) with a closed-loop structure [8]. To date, miRNAs are the most extensively studied class of small noncoding RNAs (sncRNAs) [9]; miRNAs are present in a wide range of tissues and fluids [10, 11] and play an essential role in neurological and neurodegenerative diseases by regulating cell-to-cell communication as hormone-like molecules to influence the behaviors of different cells in a paracrine or endocrine manner [12]. Compared to sncRNAs, lncRNAs are more heterogeneous in size, often polyadenylated, longer and lack open reading frames (ORFs). In early studies, the importance of lncRNAs was vastly underestimated because of their low levels of sequence conservation and expression [13]. However, accumulating evidence indicates that lncRNAs play essential roles in the development of diseases in various organisms [14]. In addition, circRNA has recently been identified as a novel type of endogenous ncRNA that is abundant yet enigmatic in mammalian cells. Unlike linear RNAs that are terminated with 5′ caps and 3′ tails, circRNAs are characterized by a covalent closed-loop structure formed by a back-splicing event, without 5′ caps or poly-A tails. Notably, one of the most frequently studied functions of circRNA is the miRNA sponge [15]. Therefore, ncRNAs have potential as candidate diagnostic biomarkers and therapeutic targets in patients with TSCI.

The regulating functions of ncRNAs after TSCI and their underlying functional mechanisms have not yet been sufficiently and systematically described. Therefore, extensive prediction and analysis of the ncRNAs regulating the progression of TSCI is fundamental for the development of understanding the underlying mechanisms and finding effective therapeutic strategies. Our study analyzed the profiles and predicted the function of differentially expressed (DE) ncRNAs in the epicenter of spinal cord lesions in a modified Allen’s weight-drop model using RNA sequencing techniques to provide a better comprehending of the diagnostic, prognostic and therapeutic value of ncRNAs.

## RESULTS

### DE ncRNAs and mRNAs

**Figure 1:**
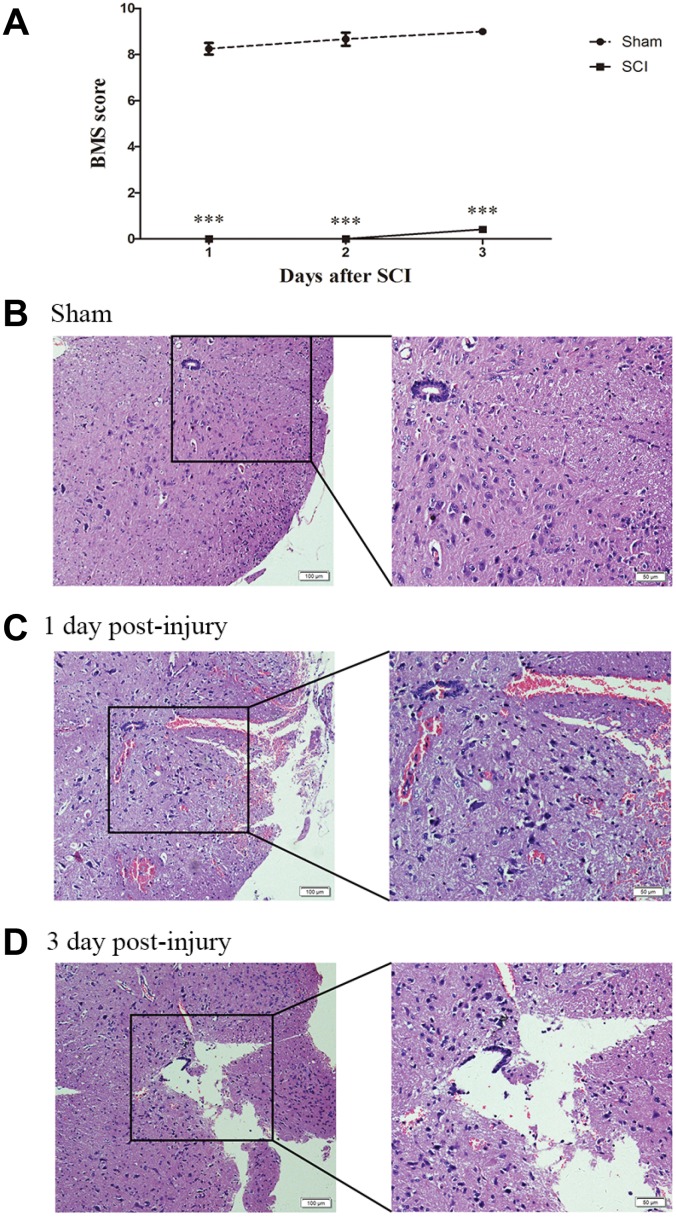
**Establishment of SCI animal model.** (**A**) BMS scores indicate the motor functional index 3 days after SCI. ***P<0.001. (**B**–**D**) H&E staining of spinal cord samples from the sham and SCI groups at days 1 and 3 postsurgery.

**Figure 2:**
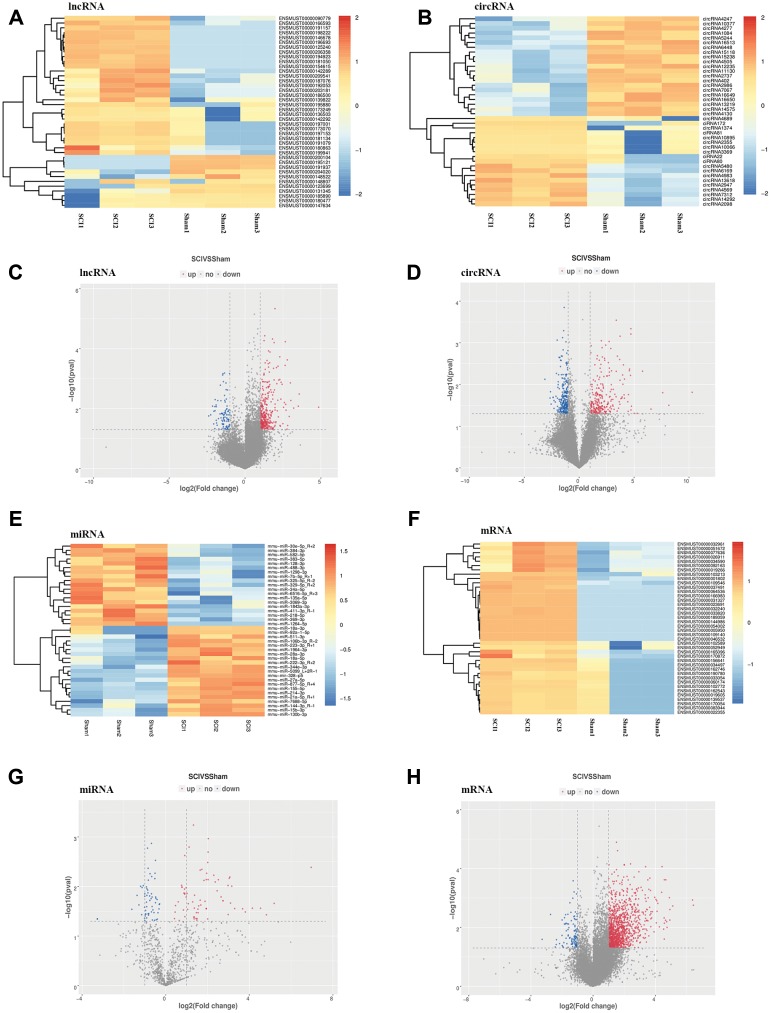
**Expression profiles of DE ncRNAs and mRNAs in the lesion epicenter after SCI.** (**A**) Heat map of DE lncRNAs in the SCI group compared with the sham group. (**B**) Heat map of DE circRNAs. (**C**) Volcano plot indicating the differential expression of lncRNAs. (**D**) Volcano plot of circRNAs. (**E**) Heat map of DE miRNAs. (**G**) Volcano plot of miRNAs. (**F**) Heat map of DE mRNAs. (**H**) Volcano plot of mRNAs. Up-regulated and down-regulated genes are colored in red and blue, respectively.

**Figure 3:**
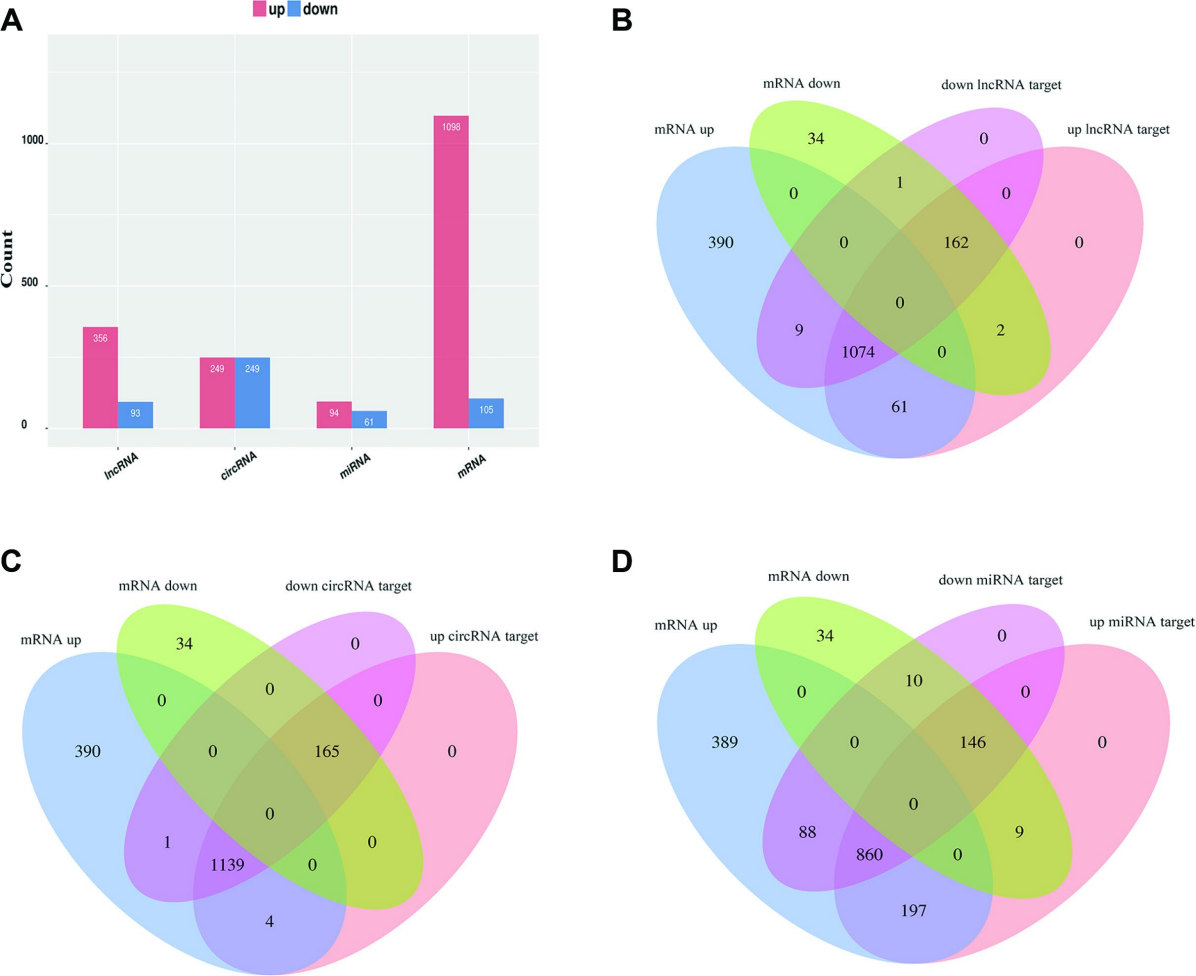
**Overview of relative differential expression of ncRNAs.** (**A**) Histogram showing the number of dysregulated ncRNAs and mRNAs. (**B**–**D**) Venn diagram showing the overlap between the target mRNAs of dysregulated ncRNAs and dysregulated mRNAs.

To identify the effect of TSCI on ncRNA expression in the lesion epicenter, we applied a standard Allen’s weight-drop model. SCI mice started to show improvements in locomotor function 2 days after TSCI, but during the first two days, the BMS was rated zero. The BMS of mice in the sham group showed an improvement on day 1 and returned to normal on day 3 postsurgery (Figure 1A). The spinal tissues of inbred C57 mice damaged by Allen’s impactor were sliced and stained with H&E. Staining results demonstrated severe damage to the blood-spinal cord barrier and the structural integrity of the lesion epicenter, including rupture, hemorrhage and inflammatory cell infiltration (Figure 1B–1D). To further determine whether ncRNAs were involved in the TSCI, the total RNA of the lesion epicenter at the T_8–10_ level was analyzed by RNA sequencing techniques. P-values <0.05 were used to assess the normalized expression of genes. The dysregulated ncRNAs and mRNAs are shown in a table, cluster map, volcano plot and Venn diagram. Information on the top 40 dysregulated circRNAs, lncRNAs, miRNAs, and mRNAs is listed in order of ascending p-value (Tables 1–4). The cluster map, volcano plot and Venn diagram of DE circRNAs, lncRNAs, miRNAs, and mRNAs after TSCI are shown in Figure 2. According to the data, we summarized the dysregulated RNAs in the TSCI samples compared with those in the sham samples, as follows: 249 circRNAs were upregulated, and 249 circRNAs were downregulated; 356 lncRNAs were upregulated, and 93 lncRNAs were downregulated; 94 miRNAs were upregulated, and 61 miRNAs were downregulated; 1098 mRNAs were upregulated, and 105 mRNAs were downregulated (Figure 3A). DE ncRNAs could directly or indirectly target genes and regulate the expression of target mRNAs. The results of an intersectional analysis of DE circRNAs, lncRNAs, and miRNAs and their target DE mRNAs are shown in a Venn diagram (Figure 3B–3D).

**Table 1:** Top 40 differently expressed miRNAs in SCI tissues comparing with Sham tissues.

**miRNA**	**Genome ID**	**Strand**	**p-value**	**log2_FoldChange**	**Regulation**
**mmu-miR-344e-3p**	chr7	−	0.000575	1.33	up
**mmu-miR-106b-3p_R-2**	chr5	−	0.001598	1.12	up
**mmu-miR-5099_L+2R-1**	chr12	+	0.002016	2.03	up
**mmu-miR-15b-3p**	chr3	+	0.002372	0.91	up
**mmu-miR-7688-5p**	chr10	+	0.003269	2.01	up
**mmu-miR-1964-3p**	chr7	+	0.004516	1.73	up
**mmu-miR-130b-3p**	chr16	−	0.004666	1.65	up
**mmu-miR-155-5p**	chr16	−	0.005421	1.96	up
**mmu-miR-27a-5p**	chr8	+	0.005929	2.30	up
**mmu-miR-18a-3p**	chr14	+	0.006440	3.07	up
**mmu-miR-18a-5p**	chr14	+	0.007264	2.03	up
**mmu-miR-223-3p_R+1**	chrX	+	0.007287	2.52	up
**mmu-miR-214-3p**	chr1	+	0.008100	2.37	up
**mmu-miR-92a-1-5p**	chr14	+	0.009857	3.16	up
**mmu-miR-28a-3p**	chr16	+	0.010636	0.98	up
**mmu-miR-877-5p_R+4**	chr17	−	0.011348	0.90	up
**mmu-miR-21a-5p_R+1**	chr11	−	0.012198	2.46	up
**mmu-miR-144-3p_R-1**	chr11	+	0.013632	0.87	up
**mmu-miR-222-3p_R+2**	chrX	−	0.013701	0.91	up
**mmu-miR-511-3p**	chr2	+	0.013759	1.02	up
**mmu-miR-369-3p**	chr12	+	0.001349	−0.68	down
**mmu-miR-384-3p**	chrX	−	0.001676	−0.87	down
**mmu-miR-325-5p_R-2**	chrX	−	0.002973	−0.49	down
**mmu-miR-34a-5p**	chr4	+	0.004289	−0.84	down
**mmu-miR-383-5p**	chr8	−	0.005397	−0.51	down
**mmu-miR-128-3p**	chr1	+	0.005516	−0.75	down
**mmu-miR-30e-5p_R+2**	chr4	−	0.006287	−0.67	down
**mmu-miR-411-3p_R-1**	chr12	+	0.006918	−0.52	down
**mmu-miR-329-5p_R+2**	chr12	+	0.007447	−0.83	down
**mmu-miR-1298-3p**	chrX	+	0.009603	−1.07	down
**mmu-miR-1264-5p**	chrX	+	0.009893	−1.20	down
**mmu-miR-135a-5p**	chr9	+	0.010159	−0.92	down
**mmu-miR-218-5p**	chr5	+	0.011109	−0.90	down
**mmu-miR-6516-5p_R+3**	chr11	+	0.011772	−0.86	down
**mmu-miR-488-3p**	chr1	+	0.013430	−0.85	down
**mmu-miR-7b-5p_R+1**	chr17	+	0.013986	−1.00	down
**mmu-miR-1843a-3p**	chr12	−	0.014024	−0.68	down
**mmu-miR-3069-3p**	chr12	−	0.015073	−1.37	down
**mmu-miR-582-5p**	chr13	−	0.015134	−0.90	down
**mmu-miR-204-5p**	chr19	+	0.015457	−0.77	down

**Table 2:** Top 40 differently expressed CircRNAs in SCI tissues comparing with Sham tissues.

**CircRNA ID**	**Chrom**	**Gene Name**	**Strand**	**p-value**	**log2_FoldChange**	**Regulation**
**circRNA8075**	chr14	Diaph3	−	0.000288	3.38	up
**circRNA81**	chr2	Thbs1	+	0.000459	4.71	up
**circRNA172**	chr11	Col1a1	−	0.000618	4.71	up
**circRNA2355**	chr4	Kif2c	−	0.000694	3.73	up
**circRNA9086**	chr5	Antxr2	−	0.000891	2.02	up
**circRNA9357**	chr4	Pgd	−	0.000930	2.73	up
**circRNA7783**	chr15	Racgap1	−	0.001137	2.42	up
**circRNA5480**	chr13	Nln	−	0.001174	1.10	up
**circRNA8879**	chr6	Zc3hav1	−	0.001308	2.09	up
**circRNA3369**	chr2	Atp8b4	−	0.001367	2.88	up
**circRNA13618**	chr11	Psmd3	+	0.001736	1.06	up
**circRNA6169**	chr11	Ankfy1	+	0.002037	1.66	up
**circRNA8690**	chr8	Gm20388	+	0.002183	2.11	up
**circRNA8894**	chr6	Skap2	−	0.002360	2.31	up
**circRNA7312**	chr16	Pak2	−	0.002420	1.62	up
**circRNA8074**	chr14	Diaph3	−	0.002507	3.69	up
**circRNA14292**	chr5	Gsap	+	0.002621	1.83	up
**circRNA7782**	chr15	Racgap1	−	0.002623	2.33	up
**circRNA8712**	chr7	Lig1	+	0.002984	2.66	up
**circRNA4569**	chr9	Fli1	+	0.003297	1.98	up
**circRNA2098**	chr4	Anp32b	+	0.003353	1.43	up
**circRNA15118**	chr1	Dpp10	−	0.000142	−1.36	down
**circRNA13219**	chr1	Pld5	+	0.002372	−1.41	down
**circRNA4130**	chr1	Pld5	−	0.000752	−1.51	down
**circRNA11130**	chr15	Lrrc6	−	0.000863	−2.00	down
**circRNA15238**	chr18	Nol4	−	0.000946	−1.20	down
**circRNA4505**	chr9	Cntn5	−	0.001476	−1.46	down
**circRNA4277**	chr18	Asxl3	+	0.001539	−1.02	down
**circRNA1084**	chr6	Dync1i1	+	0.001770	−1.13	down
**circRNA2986**	chr2	Cacna1b	−	0.002072	−1.62	down
**circRNA402**	chr9	Myrip	+	0.002090	−1.54	down
**circRNA7067**	chr17	L3mbtl4	+	0.002227	−1.83	down
**circRNA2737**	chr3	Pogz	+	0.002259	−1.76	down
**circRNA4247**	chr18	Greb1l	+	0.002611	−1.69	down
**circRNA14575**	chr4	Rimkla	−	0.002659	−1.64	down
**circRNA8251**	chr19	Cpeb3	−	0.002934	−1.13	down
**circRNA6448**	chr11	Tbc1d16	−	0.003047	−1.20	down
**circRNA16650**	chr14	Sfmbt1	+	0.003097	−1.12	down
**circRNA12235**	chr12	Dtnb	+	0.003164	−1.10	down
**circRNA16513**	chr14	Sfmbt1	+	0.003400	−1.14	down

**Table 3:** Top 40 differently expressed lncRNAs in SCI tissues comparing with Sham tissues.

**Gene id**	**Gene name**	**Status**	**p-value**	**log2_FoldChange**	**Regulation**
**MSTRG.34122**	Homez	novel	0.000005	1.98	up
**MSTRG.67284**	Tpd52	novel	0.000033	1.30	up
**MSTRG.111948**	Na	novel	0.000046	2.66	up
**MSTRG.74174**	Gm15689	novel	0.000054	1.26	up
**MSTRG.124087**	F630028O10Rik	known	0.000085	1.46	up
**MSTRG.40161**	Rbfox2	novel	0.000095	1.55	up
**MSTRG.113073**	Cfap20	novel	0.000109	1.64	up
**MSTRG.95251**	Gm44170	known	0.000109	1.80	up
**MSTRG.93249**	Na	novel	0.000159	2.29	up
**MSTRG.55738**	BE692007	known	0.000189	2.64	up
**MSTRG.52721**	Dpysl3	novel	0.000225	1.45	up
**MSTRG.60157**	Arhgap15	novel	0.000269	1.33	up
**MSTRG.33815**	Na	novel	0.000284	1.62	up
**MSTRG.25765**	Na	novel	0.000309	2.17	up
**MSTRG.77436**	Gm11216	known	0.000310	1.53	up
**MSTRG.107512**	Fgfr2	novel	0.000391	2.08	up
**MSTRG.94129**	Na	novel	0.000395	1.88	up
**MSTRG.11903**	Pcbp3	novel	0.000399	1.55	up
**MSTRG.123405**	Mamld1	novel	0.000421	1.34	up
**MSTRG.105294**	Na	novel	0.000509	1.61	up
**MSTRG.35205**	Gm26908	novel	0.000609	1.25	up
**MSTRG.14082**	Ppm1h	novel	0.000625	1.24	up
**MSTRG.33653**	NA	novel	0.000954	2.29	up
**MSTRG.33653**	Thumpd2	novel	0.000987	1.18	up
**MSTRG.83712**	AI506816	known	0.001001	2.80	up
**MSTRG.10902**	NA	novel	0.001014	1.60	up
**MSTRG.63860**	Gm14005	novel	0.001042	1.50	up
**MSTRG.110801**	Ddx60	novel	0.001081	1.47	up
**MSTRG.75978**	NA	novel	0.001084	2.21	up
**MSTRG.99804**	Kcnn4	novel	0.001140	1.92	up
**MSTRG.108182**	Tacc1	novel	0.001143	1.26	up
**MSTRG.28498**	Cenpp	novel	0.001203	1.12	up
**MSTRG.47244**	NA	novel	0.001255	1.63	up
**MSTRG.24916**	NA	novel	0.001260	2.09	up
**MSTRG.125937**	Diaph2	novel	0.001263	1.18	up
**MSTRG.98701**	3300002P13Rik	known	0.000457	−1.34	down
**MSTRG.52759**	Pcdha6	novel	0.000674	−1.45	down
**MSTRG.83528**	Pclo	novel	0.000711	−1.02	down
**MSTRG.24945**	NA	novel	0.000748	−1.47	down
**MSTRG.11329**	Rhobtb1	novel	0.001009	−1.41	down

NA, not annotated.

**Table 4:** Top 40 differently expressed mRNAs in SCI tissues comparing with Sham tissues.

**Gene id**	**Gene name**	**p-value**	**log2_FoldChange**	**Regulation**
**MSTRG.101002**	Snord34	0.000013	1.51	up
**MSTRG.80254**	Gjb3	0.000025	1.58	up
**MSTRG.104414**	P2ry6	0.000070	2.41	up
**MSTRG.114824**	Mmp3	0.000074	2.00	up
**MSTRG.65092**	H13	0.000074	1.03	up
**MSTRG.27838**	F13a1	0.000076	2.01	up
**MSTRG.13769**	Lyz2	0.000086	4.44	up
**MSTRG.121761**	Ccr1	0.000087	3.38	up
**MSTRG.20499**	Naglu	0.000129	2.95	up
**MSTRG.3656**	Ugt1a1	0.000154	1.21	up
**MSTRG.107440**	F7	0.000177	1.60	up
**MSTRG.105998**	Nupr1	0.000185	2.18	up
**MSTRG.12171**	Sbno2	0.000195	1.97	up
**MSTRG.111467**	Bst2	0.000196	2.64	up
**MSTRG.19369**	Ccl7	0.000212	3.65	up
**MSTRG.28823**	Rgs14	0.000221	1.01	up
**MSTRG.108769**	Msr1	0.000241	4.58	up
**MSTRG.117687**	Tagln	0.000241	4.68	up
**MSTRG.52513**	Cd14	0.000243	3.52	up
**MSTRG.86784**	Cxcl1	0.000244	2.26	up
**MSTRG.92027**	Flnc	0.000248	1.89	up
**MSTRG.6767**	Selp	0.000266	1.50	up
**MSTRG.31848**	Nid2	0.000272	1.27	up
**MSTRG.19493**	Ccl4	0.000275	1.37	up
**MSTRG.104483**	Folr2	0.000294	1.84	up
**MSTRG.21200**	Cd300ld	0.000325	1.72	up
**MSTRG.104929**	Adm	0.000330	1.32	up
**MSTRG.1578**	Col5a2	0.000347	1.87	up
**MSTRG.100407**	Fxyd3	0.000352	2.38	up
**MSTRG.33361**	Wdfy4	0.000387	1.01	up
**MSTRG.89616**	Lat2	0.000435	3.08	up
**MSTRG.114822**	Mmp12	0.000451	1.10	up
**MSTRG.34965**	Pbk	0.000458	2.92	up
**MSTRG.97389**	Ptpn6	0.000471	2.19	up
**MSTRG.127268**	Tmsb4x	0.000488	2.82	up
**MSTRG.20948**	Milr1	0.000501	2.23	up
**MSTRG.40133**	Ncf4	0.000508	2.51	up
**MSTRG.105902**	Il4ra	0.000509	2.79	up
**MSTRG.78174**	Elavl2	0.000260	-1.28	down

### Validation of ncRNA and mRNA expression

**Figure 4:**
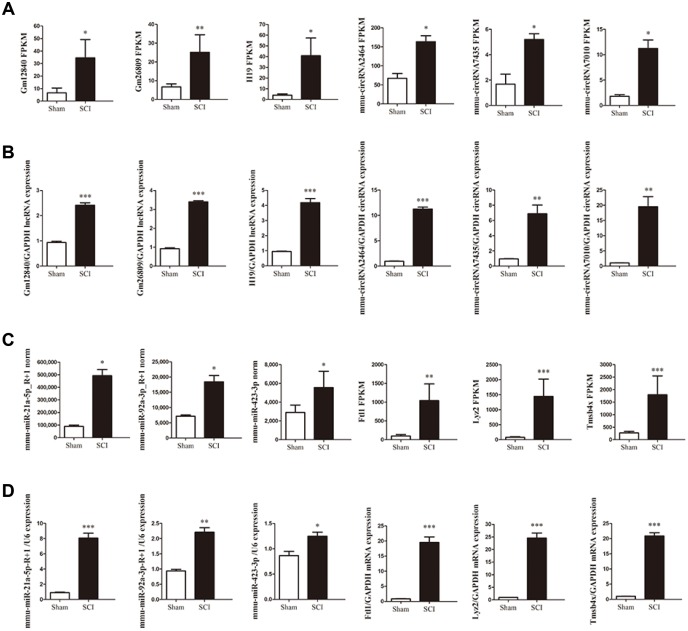
**Validation of differential ncRNA and mRNA expression.** (**A**, **C**) Sequencing results of the ncRNAs and mRNAs. (**B**, **D**) Expression of corresponding ncRNAs and mRNAs validated by qRT-PCR.

To validate the reliability of the sequencing data, the changes in the expression of 12 DE ncRNAs and mRNAs, including three circRNAs (circ2464, circ7435, circ7010), three lncRNAs (Gm12840, Gm26809, H19), three miRNAs (miR-21a-5p_-_R+1, miR-92a-3p_-_R+1, miR-423-3p) and three mRNAs (Ftl1, Lyz2, Tmsb4x) in the lesion epicenter compared with the sham group were were randomly selected for qRT-PCR analysis (Figure 4B, 4D). All the validated qRT-PCR results of the DE ncRNAs and mRNAs were consistent with the corresponding sequencing data (Figure 4A, 4C).

### Enrichment of biological functions and pathway networks

**Figure 5:**
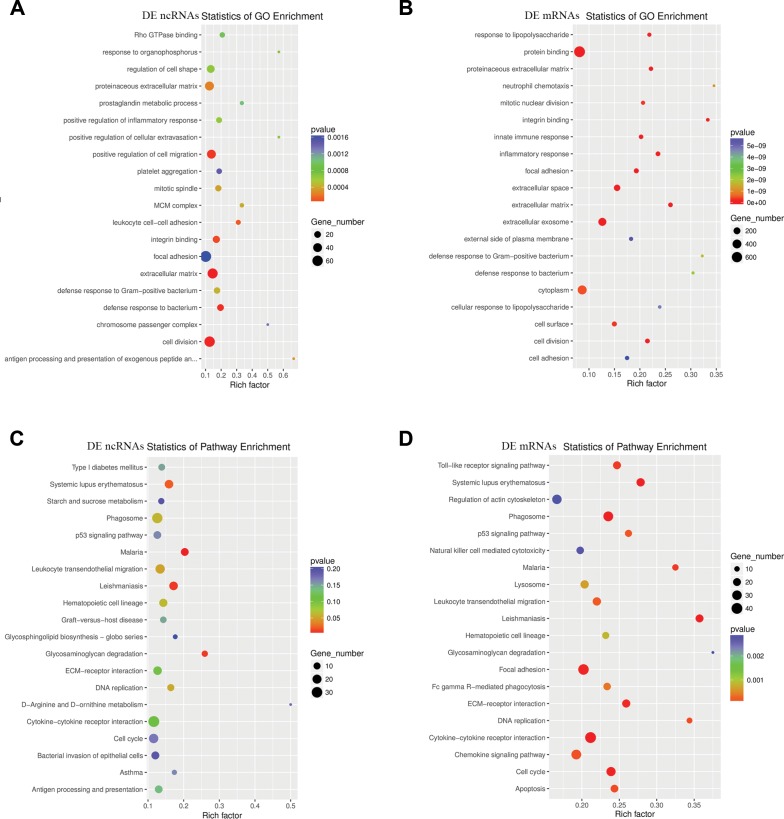
**Enriched GO terms and KEGG pathways of host genes of DE ncRNAs in SCI mice.** (**A**) Top 20 significantly enriched GO terms of DE ncRNAs are shown in the scatterplot. (**B**) Top 20 significantly enriched GO terms of DE mRNAs. (**C**) The top 20 significantly enriched KEGG pathways of DE ncRNAs are shown in the scatterplot. (**D**) The top 20 significantly enriched KEGG pathways of DE mRNAs are listed.

To detect the enrichment categories and to examine the underlying functions of ncRNAs DE after TSCI, DE ncRNAs and DE mRNAs were subjected to GO and KEGG pathway analyses. DE mRNAs and coexpressed or target mRNAs of DE ncRNAs were identified. The GO molecular function analysis showed that the dysregulated transcripts of ncRNAs were associated with cell division, focal adhesion, proteinaceous extracellular matrix, positive regulation of cell migration, extracellular matrix components, regulation of cell shape, integrin binding, defense response to bacterium and leukocyte cell-cell adhesion (Figure 5A). In addition, the significant GO items indicated that mRNAs DE after TSCI were significantly associated with cytoplasm, protein binding, extracellular exosome, extracellular space, cell surface, focal adhesion, innate immune response and mitotic nuclear division (Figure 5B). Correspondingly, the top 20 ncRNA-associated pathways were demonstrated by KEGG analysis, and the most significantly associated pathways were cytokine-cytokine receptor interaction, cell cycle, leukocyte transendothelial migration, phagosome, Leishmaniasis, Malaria and Systemic lupus erythematosus pathways, (Figure 5C). In addition, KEGG pathway analysis of DE mRNAs revealed significant associations with cytokine-cytokine receptor interaction, focal adhesion, phagosome, chemokine signaling, Regulation of actin cytoskeleton, lysosome, Cell cycle and Toll-like receptor signaling pathways, among others (Figure 5D).

### Regulatory networks of ncRNAs and mRNAs

**Figure 6:**
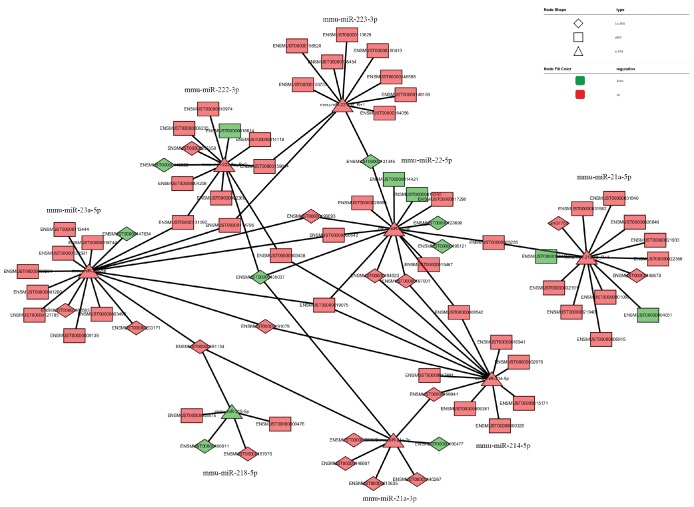
**LncRNA–miRNA–mRNA regulatory interaction network analysis.**

**Figure 7:**
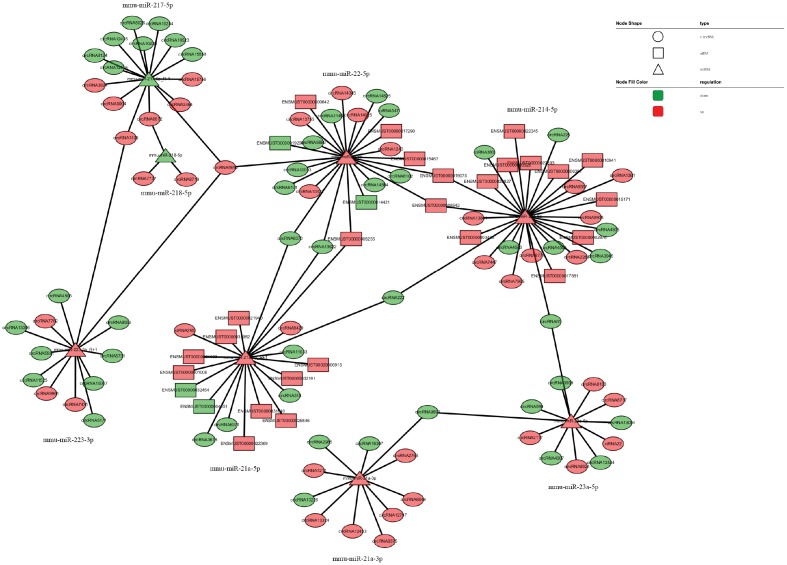
**CircRNA–miRNA–mRNA regulatory interaction network analysis.**

**Figure 8:**
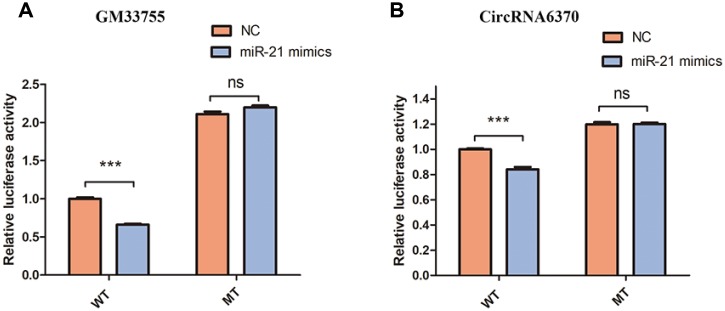
**Confirmation of the relationships.** (**A**) Relative luciferase expression of wild-type and mutant lncRNAGM33755 UTR-bearing luciferase vectors cotransfected with miR-135b expression vectors. (**B**) Relative luciferase expression of wild-type and mutant circRNA6370 UTR-bearing luciferase vectors cotransfected with miR-135b expression vectors. n=6, ***P<0.001.

The network of interactions of the host genes of these DE ncRNAs was also examined to elucidate the molecular mechanisms underlying the pathogenesis of TSCI. Considering that an important biological function of competing endogenous RNAs (ceRNAs) is binding to miRNAs, the binding relationships between ceRNAs and miRNAs were preliminarily determined. The miRNA- binding sites of lncRNAs and circRNAs were identified to construct lncRNA/circRNA–miRNA–mRNA coexpression networks. miRNA was used as the center of each network, which clearly shows possible regulated target genes. The lncRNA/circRNA–miRNA–mRNA interaction networks were conveniently displayed using Cytoscape. Eight DE miRNAs, *i.e*., miR-23a-5p, miR-222-3p, miR-223-3p, miR-22-5p, miR-218-5p, miR-214-5p, miR-21a-3p, and miR-21a-5p, and their paired ceRNAs and mRNAs were selected as intuitive examples showing parts of the whole complicated network involved in the pathogenesis of TSCI (Figures 6–7). The results show the regulatory relationship between ncRNAs and mRNAs with regard to TSCI. Furthermore, two pairs of binding relationships between ceRNAs and miRNAs were verified with a dual-luciferase reporter system. We found that the overexpression of miR-21-5p significantly decreased the luciferase activity of reporter vectors containing the wild-type lncRNA Gm33755 and circRNA6370 3′-UTR (Figure 8). Collectively, these results establish lncRNA33755 and circRNA6370 as targets of miR-21-5p.

## DISCUSSION

With the aging of the world population, increasing numbers of elderly persons are sustaining vertebral compression fractures (VCF) due to osteopenia, 25% of postmenopausal women are affected by a compression fracture during their lifetime, and spinal cord injury is one of the most serious complications of VCF in elderly patients leading to significant morbidity and mortality [16–18].

Since there are no approved therapies for restoring sensation or mobility following TSCI, achieving functional rehabilitation has been among the primary research interests of experimental neuroscientists in recent decades [19]. TSCI is a two-step process that can cause a permanent loss or reduction in bodily function below the level of the lesion site. The primary damage is the mechanical injury itself, and the secondary damage results from biochemical processes following the primary damage [3]. Physical trauma causes rupture of the blood-spinal cord barrier in the lesion epicenter, leading to hemorrhage, ischemia and inflammation, followed by local neuronal and glial cell death [5]. The nonneural damage in the lesion core ultimately resolves into a cavity surrounded by astrocytic and fibrotic scar borders [20]. Axonal regeneration is a complex procedure that includes structural synapse remodeling, axonal sprouting and regrowth across the lesion. A reduced intrinsic growth capacity, the absence of external growth stimulation and the presence of external inhibitory factors could lead to the failure of axons to regrow spontaneously across severe tissue lesions [21, 22]. The lesion compartments consist of different cell types, and cell biology influences axonal growth and regrowth in different ways [23]. Alleviating these differences is fundamental for achieving or improving axonal regeneration and designing rationally targeted interventions. Although several biomolecules are being used as diagnostic or prognostic biomarkers and therapeutic targets, they do not have sufficient accuracy or sensitivity to recognize pathogenesis, guide therapy or evaluate prognosis [5]. Since TSCI is a multifaceted pathological process, it is unlikely that any one molecule or pathway can affect the large number of obstacles that occur following trauma. Indeed, this may be the reason why many disparate treatments generate similar levels of recovery in TSCI animal models; as such, constructing the regulatory network involved with TSCI is clinically significant. In this study, we demonstrated that the expression of related ncRNAs and mRNAs significantly changes in the spinal cord tissue after traumatic injury, and we predicted the structure and potential functions of the regulatory network associated with these DE ncRNAs and mRNAs.

Recent studies have revealed the involvement of some specific miRNAs in many types of neuronal function in diseases, such as axon regrowth and neurodegeneration. MiRNAs are considered to be one of the major factors in the pathogenesis of CNS injury because of their intrinsic properties in regulating several biological functions and their potentially large impact in RNA disorders [24]. In this study, a marked dysregulating occurs in the expression of axon regrowth- or regeneration-associated mRNAs after injury, such as STAT3, p53, c-Jun, FOXO, KLFs and Sox, as well as their target DE miRNAs, miR-125b, miR-9, miR-222, miR-21, miR-135b and miR-145, respectively. In addition, miRNAs are critical regulators of the main molecular cascades regulating axonal growth, *i.e*., miR-26a and miR-222 repress the PTEN pathway, miR-124 targets the GSK-3b pathway, miR-9 targets the MAP1B–Rac1 pathway, and miR-133 inhibits the rhoA–PI3K–AKT pathway. MiRNAs also participate in inflammation, apoptosis and myelination-related lesions in neurological damage disease, *i.e*., let-7 inhibits IL-6 during inflammation, miR-29b increases proapoptotic gene expression, and miR-138 regulates myelination-related lesions.

Most strikingly, ceRNAs, which include lncRNAs, circRNAs and pseudogenic RNAs, cross-regulate each other by competing for shared miRNAs on miRNA response elements (MREs) [25]. CeRNA crosstalk is a type of posttranscriptional regulation that is mediated by miRNAs and links the functions of coding and noncoding RNAs. LncRNA can directly regulate the structure of DNA and the transcription and translation of RNA; notably, lncRNA can act as an miRNA sponge to competitively bind miRNA [14]. CircRNAs were later identified and are more enriched in neuronal tissues than other tissues because the long introns of neuronal genes promote circRNA formation [26]. The ceRNA regulation network plays a critical role in central neuropathy, i.e., the lncRNA SNHG5–KLF4–eNOS axis enhances the viability of astrocytes and microglia [27], the circRNA2837–miR-34a axis protects neurons against injury by inducing autophagy [28], and the circRNA ZNF609–miR-615–METRN axis reverses retinal neurodegeneration [29]. Recent research has even generated a complicated ceRNA network demonstrating that lncCyrano–miR-7 prevents the cytoplasmic destruction of circCdr1while repressing miR-671–circCdr1splicing in the brain [12]. In addition, to explore the roles of ncRNAs through this potential mechanism, we performed GO and KEGG pathway analysis to annotate predicted target mRNAs and predominant pathways of the differentially expressed ncRNAs. In this study, we found that the target mRNAs are involved in multiple biological processes, cellular signaling pathways, protein activities and gene splicing after SCI. Strikingly, focal adhesion was noted to be one of the most significantly enriched and meaningful terms of biological processes in both ncRNAs and mRNAs after GO analysis, and phagosome pathways was found to be one of the most significantly enriched and meaningful pathway of ncRNAs and mRNAs groups after KEGG pathway analysis.

In previous work, we demonstrated that miR-21 regulates astrogliosis through the PI3K–Akt–mTOR pathway and regulates fibrosis through the TGF-β–Smad pathway after TSCI [33, 30]. The reactive astrocytes and fibrotic scar tissue formed by perivascular cells stabilize the outer borders of the initial lesion epicenter and act as a chronic, physical, and chemical-entrapping barrier that prevents axonal regeneration [31, 32]. Our previous results suggest that miR-21 knockdown significantly suppressed scar formation and improved motor functional recovery after TSCI [30, 33]. In this article, a ceRNA regulation network with miR-21 as the center was constructed and provides initial evidence of the binding relationships between lncRNA GM33755, circRNA 6730 and miR-21. However, further specific studies are needed to determine whether lncRNA GM33755 and circRNA 6730 play roles in pathological processes after neurological injury.

Recently, our understanding of the extrinsic and intrinsic factors that block axonal regeneration or neuroplasticity has immensely improved with further illumination of the molecular events that occur following TSCI in mouse models. However, it is important to keep in mind that variations in axonal regeneration responses exist between mice and humans. In this article, we choose 3 days post-SCI as our time point, acute phase is the original point of pathological process. In acute phase, cell death and eventually axonal die back. Inflammation and axon regrowth at later time point, in sub-acute or chronic phases, then astrogliosis and fibrotic scar are done, axon struggle to regeneration. TSCI has an acute phase and a chronic phase, what should we do next is to compare different injuries severity in acute time points and chronic time points. Future efforts should be made to mimic endogenous ncRNA deregulation and determine the functions of ncRNA interactions in the context of posttranscriptional regulation as a whole. Findings arising from such studies will expand our understanding of the potential of ncRNAs, offer novel insight into the identification of new biomarkers and help guide strategies toward the development of potential therapies to enhance axonal regeneration and functional recovery during acute and chronic stages following TSCI. The spinal cord rarely repairs itself after an injury, but methods for promoting axonal regeneration are on the horizon.

## MATERIALS AND METHODS

### Mouse SCI model and experimental groups

All animal protocols were approved by the Research Ethics Committee of Shandong University (Jinan, China). All tissue samples of the SCI epicenter and parameters of Allen’s weight-drop apparatus were obtained as described in our previous study [33]. Briefly, 64 adult male C57BL/6 mice were randomly divided into the SCI (n=8)×4 and sham (n=8)×4 groups. Mice in the SCI group underwent T_8–10_ vertebral laminectomy to expose the spinal cord after anesthesia was induced with 3% pentobarbital. Then, moderate SCI was induced using a modified Allen’s weight-drop apparatus, subsequently, the muscles and skin were sutured layer by layer. Mice in the sham group underwent the same laminectomy but without SCI. The movement ability of randomly selected mice (SCI group n=3, sham group n=3) was evaluated using the Basso motor score (BMS) for 3 days. Three independent and well-trained investigators scored the animals according to the standard guidelines. Then, the final score was recorded as the average of the investigators’ scores. First batch of 16 C57BL/6 mice divided into the SCI group (n=4) and sham group (n=4), were humanely sacrificed on days 1 and 3 postsurgery, respectively, for the collection of T_8–10_ spinal cord tissues. The spinal cord lesions were analyzed after tissue samples were fixed, washed, dehydrated, cleared, embedded, frozen and stained with H&E as previously described [33]. The remaining 48 mice, SCI (n=8)×3 and sham (n=8)×3, were humanely sacrificed on day 3 postsurgery for the collection of T_8–10_ spinal cord samples, then used to extract total RNA for Sequencing and qRT-PCR validation.

### RNA library construction and sequencing, transcript abundance estimation and differential expression analysis

Total RNA of spinal specimens was extracted using the TRIzol reagent (Invitrogen, Carlsbad, CA, USA). The procedure mainly included homogenization, phase separation, RNA precipitation, washing, solubilization, and monitoring of RNA degradation. The RNA concentration and quality were measured by UV absorbance at 260/280 nm; then, a LabChip Kit (Agilent, CA, USA) was used to analyze the RNA integrity.

Small RNA single-end sequencing was performed on an Illumina HiSeq 2500 LC-BIO system (Hangzhou, China). CircRNA and lncRNA paired-end sequencing were performed on an Illumina HiSeq 4000 LC-BIO system (Hangzhou, China). The fragments per kilobase of exon per million fragments mapped (FPKM) was used to measure the relative abundance of the transcripts after aligned read files were processed by in-house scripts. The expression levels of lncRNAs and mRNAs were determined by StringTie (http://ccb.jhu.edu/software/stringtie). CIRCexplorer was used to measure the expression of circRNAs [34], unique circRNAs were generated from assemblies.

### qRT-PCR validation

As previously described [35], total RNA was reverse-transcribed into cDNA; then qRT-PCR was performed using an Applied Biosystems (Wilmington, DE, USA) 7500 RT-PCR system. GAPDH was used as an internal control to normalize relative circRNA, lncRNA and mRNA expression levels. MiRNA expression levels were normalized using U6. The 2^–ΔΔCT^ method was used for comparative quantitation. Three independent experiments were performed. The specific primers for each gene are listed in Table 5.

**Table 5:** Primers designed for qRT-PCR validation.

**Gene**	**Primer**
H19	**F** TTCACTTAGAAGAAGGTTCA
	**R** TTCCATTCTCCAGTTATTGA
Gm12840	**F** CCAAGGAGTTGACTGATTATCT
	**R** ACACAAGCAAGACCAATACA
Gm26809	**F** ATCTCTAAGCACACTCGTCCAC
	**R** ACTAATCGCCGCCGTCAG
circRNA7010	**F** CTGGAGACTGTGGAAAGC
	**R** TGTAAGGACACTGGGGC
circRNA2464	**F** CTGTCAAGTATGTGGAGTG
	**R** CAACAGCACCATCACC
circRNA7435	**F** ATGACATCCGCAGAAGG
	**R** AGGCAAATACCGCACTC
mmu-miR-21a-5p_R+1	**F** CGGGCGTAGCTTATCAGACTG
	**RT** GTCGTATCCAGTGCAGGGTCC
	GAGGTATTCGCACTGGATACGACGTCAAC
mmu-miR-423-3p	**F** TTAGCTCGGTCTGAGGCCC
	**RT** GTCGTATCCAGTGCAGGGTCC
	GAGGTATTCGCACTGGATAC
	GACACTGAG
mmu-miR-92a-3p	**F** CCGTATTGCACTTGTCCCG
	**RT** GTCGTATCCAGTGCAGGGTCC
	GAGGTATTCGCACTGGATAC
	GACACAGGC
Tmsb4x	**F** AGAACTACTGAGCAGGAAGG
	**R** GGACATCTTTGACCATCTTGAA
Lyz2	**F** ATGAAGACTCTCCTGACTCTG
	**R** ATAGTAGCCAGCCATTCCAT
Ftl1	**F** TGGAGAAGAACCTGAATCA
	**R** AGGAAGTCACAGAGATGAG
GAPDH	**F** GGTGAAGGTCGGTGTGAACG
	**R** CTCGCTCCTGGAAGATGGTG
U6	**F** CTCGCTTCGGCAGCACATATACT
	**R** ACGCTTCACGAATTTGCGTGTC

### GO annotations and KEGG pathway analysis

GO annotations and KEGG pathway analysis were performed to investigate the potential roles of all DE ncRNAs. GO analysis includes three domains, cellular components, biological processes, and molecular functions, and provides a controlled vocabulary to describe DE mRNAs (P<0.05) in GO categories (http://www.geneontology.org) [36]. In addition, the KEGG database (http://www.genome.ad.jp/kegg/) was used to detect the potential functions of the target genes in the identified pathways [37], with significance indicated by P-values <0.05.

### Analysis of ncRNA regulatory network

An ncRNA regulatory network was constructed to examine the interactions and functional links among dysregulated mRNAs and ncRNAs in the pathological process of SCI. The target mRNAs of miRNA were predicted by software programs as previously described [35]. We selected the dysregulated target RNAs correlating to DE ncRNAs. Cytoscape software (San Diego, CA, USA) was used to construct interaction networks for lncRNA–miRNA–mRNA and circRNA–miRNA–mRNA.

### Luciferase assay

293T cells were cultured in 94-well plates and cotransfected with luciferase reporter constructs containing lncRNA GM33755 or circRNA 6370 (LC) and a Renilla luciferase construct (Invitrogen), miRNA-21 mimic or scrambled negative control (LC) were transfected using Lipofectamine 2000 (Invitrogen) for 6 h. After 48 h of culture at 37°C, the culture supernatant was mixed with LAR II and measured using an illuminometer. Then, a luciferase activity assay was performed using a dual luciferase reporter system (E1910, Promega, Madison, WI, USA). In addition, Stop&Glo Reagent used as an internal control. The results shown represent the means of three experiments and are presented as the mean ± standard deviation (SD).

### Statistical analysis

SPSS 20.0 (IBM, Chicago, IL, USA) and GraphPad Prism software (La Jolla, CA, USA) were used to perform the statistical analysis. Data are presented as the mean ± SD. ANOVA and Student’s t-test were used for comparisons (P<0.05). The Chi-squared 2X2 test, Chi-squared nXn test and Fisher’s exact test were used to assess the differential expression of miRNA (P<0.05), differential lncRNAs expression was examined using the R package Ballgown (P<0.05) and CircRNA expression in the different samples and groups was calculated using scripts developed in-house (P<0.05).

### Ethical disclosure

The authors state that the study was approved by the Ethics Committee of Jinan Central Hospital Affiliated to Shandong University.
